# Delayed Presentation of Pharyngeal Erosion after Anterior Cervical Discectomy and Fusion

**DOI:** 10.1155/2015/173687

**Published:** 2015-01-29

**Authors:** Amit Nathani, Alexander E. Weber, Trevor C. Wahlquist, Gregory P. Graziano, Paul Park, Rakesh D. Patel

**Affiliations:** ^1^Department of Orthopaedic Surgery, University of Michigan, Ann Arbor, MI 48109, USA; ^2^Department of Neurosurgery, University of Michigan, Ann Arbor, MI 48109, USA

## Abstract

Dysphagia after anterior cervical discectomy and fusion (ACDF) is common, with a prevalence ranging between 28% and 57% of cases. However, nearly all cases resolve spontaneously within 2 years, thus identifying patients who require more detailed or invasive work-up is a challenging task for clinicians. A review of literature reveals a paucity of case reports detailing work-up and successful management options. The authors performed a clinical and radiographic review of a case of a 47-year-old female who presented with persistent dysphagia 3 years following anterior cervical spine surgery and was found to have an erosive pharyngeal defect with exposed spinal hardware. The diagnosis was made with direct laryngoscopy and treatment consisted of plate removal and pharyngeal repair, followed by revision fusion with deformity correction. This case and the accompanying pertinent review of the literature highlight the importance of a thorough evaluation of dysphagia, especially in the mid- and late-term postoperative period following ACDF, when most cases of dysphagia should have been resolved. Correctly identifying the underlying etiology of dysphagia may lead to improved revision of ACDF outcomes. Unresolved dysphagia should be a red flag for surgeons as it may be the presentation of erosive esophageal/pharyngeal damage, a rare but serious complication following ACDF.

## 1. Introduction

Esophageal injuries are a rare complication of anterior cervical spine surgery [1–3]. The vast majority of esophageal injuries occur intraoperatively from sharp instrumentation or retraction, are repaired acutely at the time of injury, and typically heal reliably without subsequent clinical sequelae [4]. Delayed esophageal perforations, however, are much less common and the available literature shows etiologies ranging around graft extrusion, plate migration, or screw pullout or loosening and is associated with significantly higher morbidity and one reported case of fatality [1, 3, 5]. Diagnosis is difficult because postoperative dysphagia after anterior cervical spine surgery is common, ranging between 28% and 57%. However, nearly all cases resolve without intervention, and less than 2% of patients report persistent dysphagia at 2-year follow-up [6–8]. We report a patient who underwent anterior cervical discectomy and fusion (ACDF) and experienced plate migration and erosion through the posterior pharyngeal wall. Their presentation highlights the need for critical evaluation of the patient reporting persistent dysphagia even remotely after anterior cervical surgery, as rare and potentially fatal complications that are correctable could be missed.

## 2. Case Report

The patient was a 47-year-old female with a history of rheumatoid arthritis who presented to our institution with dysphagia and neck pain after multiple previous cervical spine surgeries at an outside hospital. Her history began with an ACDF from C5 to C7 in 2009 for neck and arm pain. Following her index procedure, she experienced nonunion with loosening of screws and thus underwent a revision extension ACDF from C3 to C7 in 2010 which resulted in significant and immediate neck and arm pain relief. Despite her pain relief, her postoperative course was complicated by superficial surgical site infection necessitating irrigation and debridement 3 days later and antibiotics. Despite no apparent intraoperative complications or oropharyngeal perforation, she experienced significant dysphagia following the third surgery, and, despite unremarkable serial esophagrams, required a feeding tube for approximately 1 year. Gradually, after this period of oropharyngeal rest, her dysphagia somewhat improved and she was able to tolerate a mechanical soft diet with some difficulty. The patient had sustained pain relief but persistent dysphagia for approximately 3 years without any additional work-up. The patient initially presented to our institution following a low-energy fall. Her symptomatology had not changed but routine cervical spine imaging was obtained and she was subsequently referred to our spine specialty clinic for continued evaluation.

**Figure 1:**
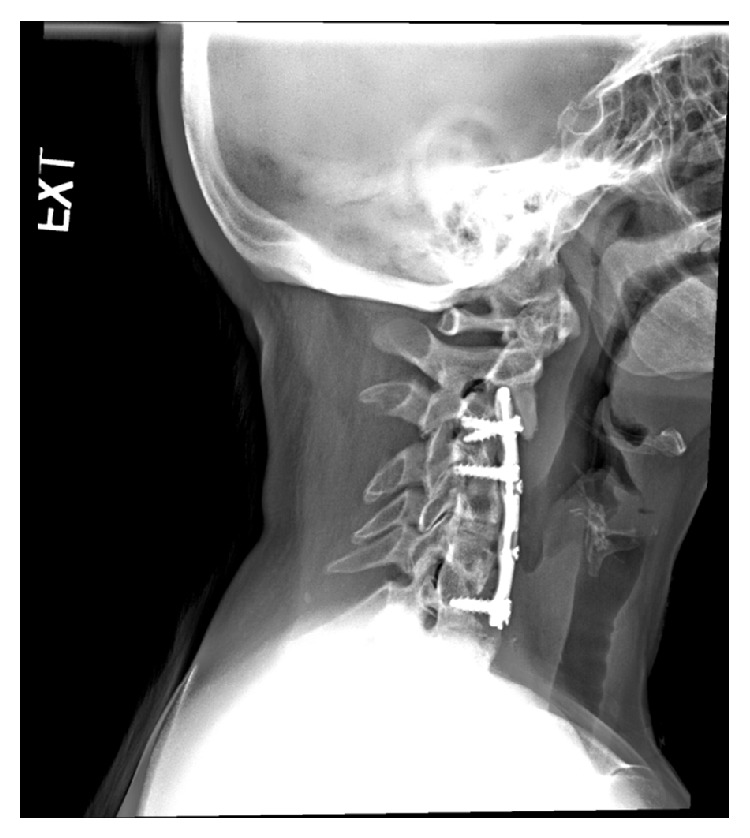
Plain radiograph, lateral view, of the cervical spine demonstrates air that appears to be communicating with plate.

**Figure 2:**
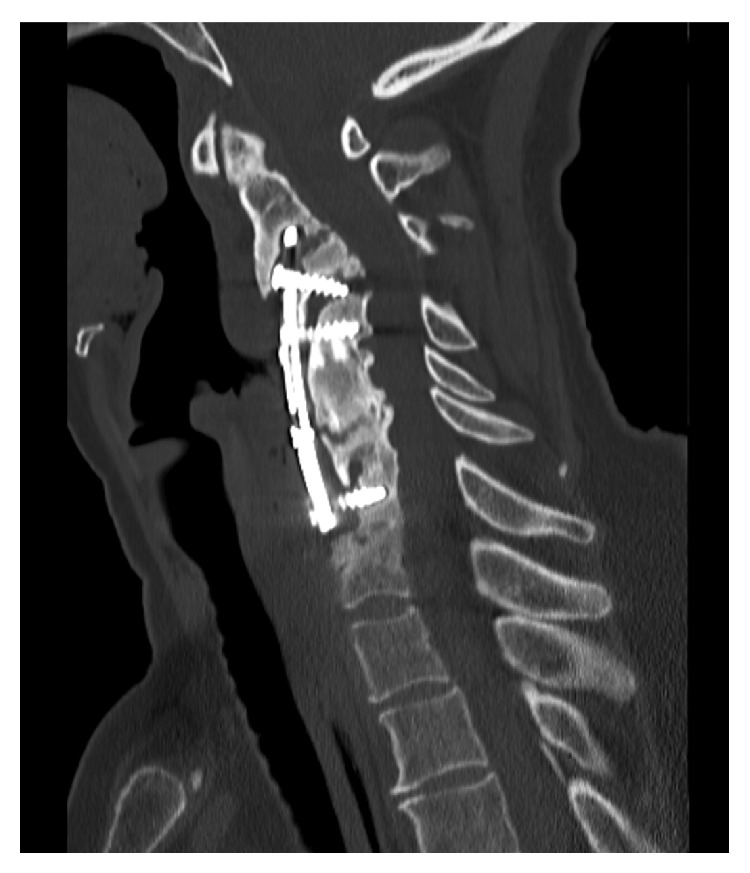
Sagittal CT view shows evidence of pseudarthrosis, lucency around screws at C3 and C7, and anterolisthesis of C2 in relation to C3, with the C2 vertebra overriding the superior aspect of the metallic plate.

At the time of her presentation, her plain film radiography (Figure 1) and computed tomography (CT) of the cervical spine (Figure 2) revealed nonhealing posterior pseudarthrosis with local kyphosis, screw loosening, plate migration, and air communicating with the plate raising concern for esophageal perforation. In the context of her persistent dysphagia, mild pain, and imaging findings, it was deemed reasonable to proceed with neck exploration in conjunction with our otolaryngology colleagues with plans to remove hardware and then perform staged revision extension of previous fusion from a posterior approach.

**Figure 3:**
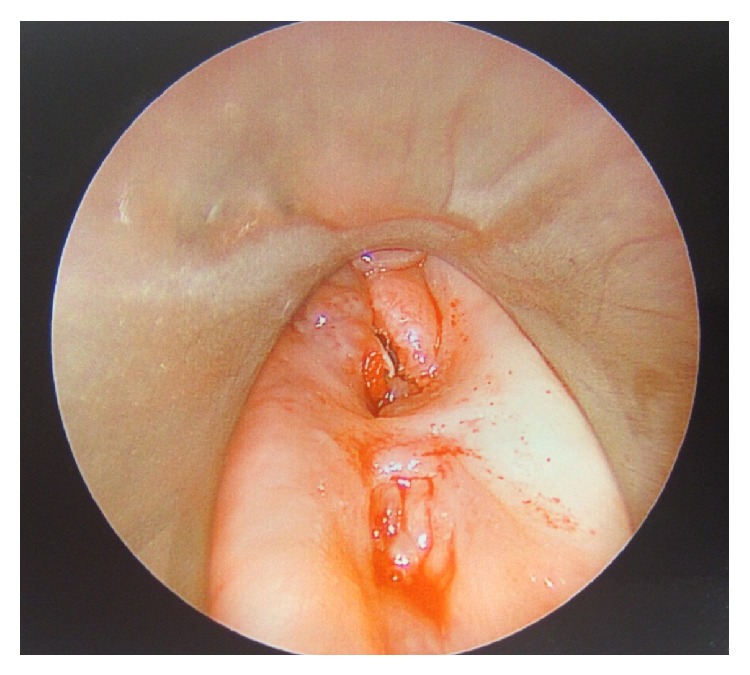
Direct laryngoscopy with telescopic visualization of pharyngeal defect with exposed hardware.

The patient was brought to the operating room and direct laryngoscopy was performed. A pharyngeal defect with exposure of the spinal hardware was noted in the posterior pharynx in the midline (Figure 3). Further evaluation beyond the proximal esophagus was not attempted for fear of worsening the defect. A nasogastric tube was inserted and attention was turned to the neck dissection.

The previous left vertical incision was incorporated into the planned surgical incision, as is customary with revision cervical procedures to protect the contralateral recurrent laryngeal nerve, and a standard anterior approach to the cervical spine was undertaken. The dissection was maintained above the level of the inferior aspect of the thyroid cartilage therefore minimizing risk to the recurrent laryngeal nerve, which was monitored with electromyography throughout the case. The cervical plate was found to have eroded through a 2.5 cm posterior pharyngeal wall defect that appeared to be chronic. Reconstruction of the pharynx was performed after plate removal by oversewing the defect with imbricating stitches. The decision was then made to prepare for a staged procedure with posterior spinal fusion (PSF) in the immediate future, but, first, a C3-C4 cervical osteotomy was performed to remove any anterior bony elements and allow deformity correction during definitive fusion. A drain was left in place, and the patient was placed in a halo for temporary cervical spine stabilization.

Two days following the initial surgery, the patient was again taken to the operating room and underwent posterior cervical osteotomies and facet resections at C2-C3 and C3-C4 and PSF from C1 to T2 with correction of kyphotic deformity. Her postoperative course consisted of no intake by mouth with nutrition through Dobhoff feeding tube. The halo was left on for a total of 1 month postoperatively and then removed. She remained in a skilled nursing facility for 1 month postoperatively. The patient's pain and dysphagia improved, and there was no evidence of frank contrast extravasation on the immediate or 2-month follow-up swallow study. Flexible fiber optic laryngoscopy at those time intervals also demonstrated an upper airway in continuity without evidence of erosion or abnormalities. The patient's diet was advanced to a mechanical soft diet at 2 months postoperatively, which she tolerated well, and the patient self-advanced her diet to a regular diet approximately 6 months postoperatively with some mild episodic dysphagia. Flexible fiber optic laryngoscopy at 8 months postoperatively demonstrated a well-healed posterior pharyngeal wall all the way to the cricopharyngeus without evidence of exposed cervical spine or cervical spine hardware or any other visual abnormality.

## 3. Discussion

Soft tissue injuries are a well-recognized complication of the anterior approach to the cervical spine. The incidence of esophageal perforation from all causes with and without instrumentation ranges between 0.25% and 1.49% based on three large retrospective reviews by Fountas and colleagues [1] of 1,015 patients, Newhouse et al. [3] of 10,000 patients, and Gaudinez et al. [2] of 2,946 patients. The Cervical Spine Research Society described hardware failure as the third most common cause of esophageal perforation after esophageal retraction and esophageal manipulation intraoperatively [9]. The vast majority of those perforations occur acutely during the index operation due to retraction or sharp instrumentation, with very few cases in the literature occurring as a late complication. The late complications reported in the literature typically present with more dramatic clinical deterioration such as recurrent pneumonias, cervical abscesses, sepsis, mediastinitis, meningitis, and unexplained fevers [2, 5, 10]. Typically, when this complication occurs late, the most common cause is hardware failure, ranging between 9% and 35%, but the sample size is low. Previous studies have demonstrated a positive correlation between the length of the fusion and the risk of hardware failure [11, 12].

In contrast to esophageal injuries, dysphagia after anterior cervical surgery is quite common, with varying etiologies and an increased incidence with multiple-level fusions and no increased association noted with instrumentation compared to noninstrumented fusion [13]. Dysphagia, however, follows a transient course, which further complicates which subset of patients requires further work-up and when to complete this work-up if dysphagia does not resolve. Eroğlu and colleagues [14] showed delays in both diagnosis and treatment significantly increasing the risks of morbidity and mortality, and work-up should be completed as soon as clinically suspected. In one series, upper esophageal injury carried 20% mortality in the first 24 hours, which then increased to 50% in subsequent days [15]. In our case, a low-energy fall prompted routine plain films taken 3 years following her index procedure, and she was then referred to our specialty clinic. Our interpretation of her imaging was concerning both adjacent segment disease and demonstrating a small component of air communicating with the plate construct, triggering a more detailed work-up. While it is certainly possible that her fall could have caused hardware failure and pharyngeal perforation, we feel it is unlikely the fall caused her hardware failure and pharyngeal perforation, as her symptoms of pain and dysphagia before and after the fall were unchanged. It is more likely that her erosive defect had been present for some time and causative of her dysphagia, despite the lack of a more dramatic clinical deterioration or presentation such as abscess or sepsis. In this case, radiographs prompted further detailed work-up; however, it is important to note that Han and colleagues [11] showed that a negative radiographic exam does not rule out esophageal injury and most diagnoses are based on clinical symptoms.

Conservative treatment versus operative intervention for esophageal perforation during anterior cervical spine surgery is still controversial and should be approached on a case-by-case basis. Certainly esophageal perforation noted intraoperatively should be repaired compared to delayed presentation cases. Proponents of operative treatment cite an abscess rate from 25% to 45% with nonoperative management [16]. Operative management is best accomplished with a multidisciplinary team. Choices for esophageal repair include primary repair with and without soft tissue reinforcement from an omental free flap or a pectoralis, longus colli, or sternocleidomastoid (SCM) muscle flap. Benazzo et al. [17] described the SCM muscle flap technique, and Ahn and colleagues [18] showed good results in three cases, considering it the preferable technique over the omental free flap due to its proximity to the cervical esophagus, ease in mobilization, size, and decreased chance of microvascular thrombosis [19].

## 4. Conclusion

We report a case of pharyngeal perforation after ACDF in a patient with persistent dysphagia several years after index and revision surgery. In our patient, there was no change in the patient's symptomatology; however, a recent fall prompted routine imaging and further work-up revealed adjacent segment disease and also raised concern for esophageal injury as the etiology of her dysphagia. Most esophageal perforations are noted at the time of surgery or in the immediate postoperative setting. While delayed presentations have been reported, they typically present with dramatic clinical deterioration and carry a mortality risk between 20% and 50%. When clinically suspected, prompt diagnosis and treatment are mandatory and a negative radiographic exam does not rule out esophageal injury. Operative fixation is recommended, and there are a variety of options. Most authors advocate plate removal and immediate repair of the defect. Controversy remains regarding the approach to cervical stability and whether that stabilization should be delayed or provided definitively at the time of esophageal repair. Intraoperatively, plate removal, defect repair, and assessment of subsequent cervical stability should be performed. In our case, cervical stability following plate removal and defect repair was compromised and local kyphotic deformity and pseudarthrosis prompted staged PSF. Patients who sustain esophageal perforations that are recognized and repaired after anterior cervical spine surgery tend to recover well, but, if missed, these perforations can have devastating complications. Even in cases of subtle symptomatology, persistent dysphagia 2-3 years following ACDF should prompt a more thorough work-up, as these symptoms may be an indication of underlying esophageal/pharyngeal pathology, which, if missed, can lead to significant morbidity and mortality.

## References

[1] Fountas K. N., Kapsalaki E. Z., Smith B. E. (2007). Interobservational variation in determining fusion rates in anterior cervical discectomy and fusion procedures. *European Spine Journal*.

[2] Gaudinez R. F., English G. M., Gebhard J. S., Brugman J. L., Donaldson D. H., Brown C. W. (2000). Esophageal perforations after anterior cervical surgery. *Journal of Spinal Disorders*.

[3] Newhouse K. E., Lindsey R. W., Clark C. R., Lieponis J., Murphy M. J. (1989). Esophageal perforation following anterior cervical spine surgery. *Spine*.

[4] Crowder T. T., Fischgrund J. D. (2011). Cervical radiculopathy: anterior surgical approach. *Rothman-Simeone, The Spine*.

[5] Lu D. C., Theodore P., Korn W. M., Chou D. (2008). Esophageal erosion 9 years after anterior cervical plate implantation. *Surgical Neurology*.

[6] Edwards C. C., Karpitskaya Y., Cha C. (2004). Accurate identification of adverse outcomes after cervical spine surgery. *The Journal of Bone and Joint Surgery. American Volume*.

[7] Lee M. J., Bazaz R., Furey C. G., Yoo J. (2007). Risk factors for dysphagia after anterior cervical spine surgery: a two-year prospective cohort study. *Spine Journal*.

[8] Smith-Hammond C. A., New K. C., Pietrobon R., Curtis D. J., Scharver C. H., Turner D. A. (2004). Prospective analysis of incidence and risk factors of dysphagia in spine surgery patients: comparison of anterior cervical, posterior cervical, and lumbar procedures. *Spine*.

[9] Kelly M. F., Spiegel J., Rizzo K. A., Zwillenberg D. (1991). Delayed pharyngoesophageal perforation: a complication of anterior spine surgery. *Annals of Otology, Rhinology and Laryngology*.

[10] Welsh L. W., Welsh J. J., Chinnici J. C. (1987). Dysphagia due to cervical spine surgery. *Annals of Otology, Rhinology & Laryngology*.

[11] Han S. Y., McElvein R. B., Aldrete J. S., Tishler J. M. (1985). Perforation of the esophagus: correlation of site and cause with plain film findings. *American Journal of Roentgenology*.

[12] Vaccaro A. R., Falatyn S. P., Scuderi G. J. (1998). Early failure of long segment anterior cervical plate fixation. *Journal of Spinal Disorders*.

[13] Bazaz R., Lee M. J., Yoo J. U. (2002). Incidence of dysphagia after anterior cervical spine surgery: a prospective study. *Spine*.

[14] Eroğlu A., Kürkçüoğlu I. C., Karaoğlanoğlu N., Tekinbaş C., Yimaz Ö., Başoğlu M. (2004). Esophageal perforation: the importance of early diagnosis and primary repair. *Diseases of the Esophagus*.

[15] Jones W. G., Ginsberg R. J. (1992). Esophageal perforation: a continuing challenge. *Annals of Thoracic Surgery*.

[16] Patel N. P., Wolcott W. P., Johnson J. P. (2008). Esophageal injury associated with anterior cervical spine surgery. *Surgical Neurology*.

[17] Benazzo M., Spasiano R., Bertino G., Occhini A., Gatti P. (2008). Sternocleidomastoid muscle flap in esophageal perforation repair after cervical spine surgery: concepts, techniques, and personal experience. *Journal of Spinal Disorders and Techniques*.

[18] Ahn S.-H., Lee S.-H., Kim E. S., Eoh W. (2011). Successful repair of esophageal perforation after anterior cervical fusion for cervical spine fracture. *Journal of Clinical Neuroscience*.

[19] Reid R. R., Dutra J., Conley D. B., Ondra S. L., Dumanian G. A. (2004). Improved repair of cervical esophageal fistula complicating anterior spinal fusion: free omental flap compared with pectoralis major flap. Report of four cases. *Journal of Neurosurgery*.

